# Factors That Influence the Judgment of Oral Management Necessity in Preoperative Oral Screening

**DOI:** 10.3390/ijerph182212236

**Published:** 2021-11-22

**Authors:** Nanami Kai, Yoko Tsukamoto, Kaoru Urabe, Asuka Tani, Yuko Inai, Asako Okadome, Haruhiko Kashiwazaki, Shinsuke Mizutani, Naohisa Wada

**Affiliations:** 1 Department of Medical Technology, Kyushu University Hospital, Kyushu University, 3-1-1 Maidashi, Higashi-ku, Fukuoka 812-8582, Japan; kai.nanami.516@m.kyushu-u.ac.jp (N.K.); taniguchi.yoko.836@m.kyushu-u.ac.jp (Y.T.); yamashita.kaoru.817@m.kyushu-u.ac.jp (K.U.); okadome.asako.727@m.kyushu-u.ac.jp (A.O.); 2 Section of Geriatric Dentistry and Perioperative Medicine in Dentistry, Division of Maxillofacial Diagnostic and Surgical Sciences, Faculty of Dental Science, Kyushu University, 3-1-1 Maidashi, Higashi-ku, Fukuoka 812-8582, Japan; atani@dent.kyushu-u.ac.jp (A.T.); kashi@dent.kyushu-u.ac.jp (H.K.); 3 Division of General Dentistry, Kyushu University Hospital, Kyushu University, 3-1-1 Maidashi, Higashi-ku, Fukuoka 812-8582, Japan; iyuko@dent.kyushu-u.ac.jp; 4 OBT Research Center, Faculty of Dental Science, Kyushu University, 3-1-1 Maidashi, Higashi-ku, Fukuoka 812-8582, Japan; 5 Department of General Dentistry, Division of Interdisciplinary Dentistry, Faculty of Dental Science, Kyushu University, 3-1-1 Maidashi, Higashi-ku, Fukuoka 812-8582, Japan; wada@dent.kyushu-u.ac.jp

**Keywords:** perioperative period, oral care, retrospective study

## Abstract

Oral management during the perioperative period is important to prevent the development of postoperative complications. However, there are no unified systems to examine the oral status of patients and very few studies have focused on preoperative oral screening. In this study, we examined the oral status of patients who underwent oral screening at a University Hospital. A total of 1173 patients who underwent oral screening for perioperative management from April 2020 to July 2021 were enrolled. The subjects’ medical data were retrospectively extracted from the dental records, and finally, the data of 1081 patients aged ≥20 years were analyzed. Oral screening based on seven categories was performed by dentists or dental hygienists. Our cumulative results determined whether patients required oral management during the perioperative period. “Poor oral hygiene” was the most frequent category (24%) of all oral categories examined. Logistic analysis revealed that tooth mobility had the highest odds ratio (21.476; 95% confidence interval: 11.462–40.239; *p* < 0.001) for oral management necessity during the perioperative period. Our study suggests that poor oral hygiene is most frequently observed in preoperative oral screening. Moreover, tooth mobility in preoperative oral screening may influence the judgment of oral management necessity during the perioperative period.

## 1. Introduction

Oral management during the perioperative period is considered important, mainly as a step toward preventing the development of postoperative complications, such as postoperative pneumonia, surgical site infection, and dental injury associated with anesthesia [1,2,3,4]. Moreover, recent review reports of patients undergoing total hip or knee arthroplasty with local anesthesia revealed that oral health assessment is important to ensure the elimination of oral infectious sources before elective joint arthroplasty [5]. During perioperative oral management, dentists remove the source of infection via procedures such as tooth extraction and periodontal treatment before surgery; dental hygienists educate patients to improve the oral health with an emphasis on motivating self-care. In addition, oral hygiene management and the recovery of oral function, including the swallowing function, are conducted during the perioperative period [6,7,8,9].

In Japan, perioperative oral management was introduced into the Japanese universal health insurance system in 2012 [10]. Initially, insurance coverage was ensured for patients with cancer, organ transplantation, and cardiovascular surgery. Presently, the adaptation process has been expanded to also include patients with the following applicable surgeries: (1) surgery for malignant tumors in the head and neck, respiratory, and digestive areas; (2) cardiovascular surgery; (3) orthopedic surgery, such as total hip arthroplasty; (4) organ transplant surgery; (5) hematopoietic stem cell transplant; and (6) surgery for stroke. However, this perioperative oral management system was employed only when the surgeon or the anesthesiologists in charge requested oral management before the operation. Therefore, not all patients undergoing surgery received perioperative oral management. In other words, even in case of a problem in the oral cavity, patients may undergo surgery without being examined for oral cavity by a dentist or dental hygienist.

The number of surgeries has increased with aging and the application of minimally invasive surgical methods, which has made it difficult to evaluate the oral status of all patients undergoing surgery. In addition, if all patients who do not require dental treatment visit dentists, it may contribute to the increase in medical costs and burden on patients [11]. Unfortunately, there are only a few studies that have focused on preoperative oral screening tools. We hypothesized that there are many important oral conditions which should be considered preoperatively. The present study aimed to examine the available data on the oral status of patients who undergo preoperative oral screening to determine the factors associated with the decision of undertaking preoperative oral screening for the further development of useful oral screening tools.

## 2. Materials and Methods

### 2.1. Participants and Study Design

This study included 1173 subjects who had received an oral screening for perioperative management between April 2020 and July 2021 at the Kyushu University Hospital. The subjects underwent a face-to-face oral screening by well-trained dentists or dental hygienists on the day when the surgical policy was established. The results of screening were retrospectively extracted from the dental records. The exclusion criterion for the subjects was age <20 years (*n* = 92), and the data from 1081 patients were analyzed.

The Perioperative Oral Care Center was established in this hospital in 2014; however, the targets were limited to fixed clinical department patients, and there were no systems to check the oral status of all patients who underwent surgeries. Therefore, this center initiated preoperative oral screening by dentists and dental hygienists in April 2020. Currently, the targets for oral screening are patients from the departments of gynecology, dermatology, orthopedic surgery, plastic surgery, and ophthalmology. Patients with oral management necessity are recommended to visit their family dentistry clinic or dentists at a university hospital.

The design of the present study was approved by the Kyushu University Institutional Review Board for Clinical Research (Approval No. 21021-00). The purpose of this study and the method of requesting exclusion from the study were posted on the university hospital website and the homepage of departments. A sufficient opt-out period for the patients was accordingly set up. The analysis was performed using anonymized data to allow individuals to be identified.

### 2.2. Preoperative Oral Screening Method

First, patients received an explanation of the importance of oral management during the perioperative period. After verbal agreement for screening, they were asked whether they had any subjective symptoms in the oral cavity. Moreover, they were asked if there was a family dentist to maintain the oral status or to visit in case of any oral troubles. The oral screening was conducted as depicted below, and each oral examination category was evaluated to check for applicability. Finally, the dentists comprehensively determined if any oral problems needed to be addressed during the perioperative period.

Because the study was conducted in accordance with the COVID-19 guidelines, we took sufficient infection control measures and made a decision as quickly as possible.

#### 2.2.1. Tooth Mobility

A tooth with mobility may be damaged by force at the time of intubation [4,12]. To evaluate the extent of tooth mobility, Miller’s classification was applied [13]. All teeth in the oral cavity were examined, and classes 2 and 3 were judged to be problematic.

#### 2.2.2. Isolated Tooth

The presence of isolated teeth, with no teeth on either side, was investigated, considering that it is relatively more susceptible to traumatic force during tracheal intubation [4].

#### 2.2.3. Severe Dental Caries

Tooth decay can be sharp and damage the intubation tube. These teeth can also be broken during intubation. In addition, it can cause tooth pain or dental infection, which may affect the general health condition [14]. The examiner evaluated the tooth with severe dental caries such as tooth stump and tooth fracture using a hand light for inspection with reference to the Japanese version of the Oral Health Assessment Tool (OHAT-J) [15].

#### 2.2.4. Tooth Pain

Tooth pain can adversely affect postoperative early oral intake through masticatory disorders [14]. The patients were asked by the examiner, “Do you have any tooth pain?”

#### 2.2.5. Inadaptation of Denture

Not only chewing disorders but also inadaptation of partial dentures can cause damage at the time of intubation [16]. If the patients have dentures, they were asked if they experience any pain owing to the dentures. The examiners also evaluated if dentures fall or lift when opening.

#### 2.2.6. Gingival Inflammation

The category “ums and tissues” in OHAT-J was used [15] (Table 1). If the score was 1 or 2, patients were diagnosed with gingival inflammation.

#### 2.2.7. Oral Dryness

Severe oral dryness compromises taste, swallowing, digestion, and nutrition [14]. The category “Saliva” in OHAT-J was used in this study [15] (Table 1). If the score was 2, the patients were diagnosed with oral dryness.

#### 2.2.8. Poor Oral Hygiene

Poor oral hygiene, including that of the tongue, can affect postoperative pneumonia or fever [7,17]. The category “Oral cleanliness” in OHAT-J was used for this test [15] (Table 1). If the score was 1 or 2, patients were diagnosed with poor oral hygiene.

### 2.3. Statistical Analysis

First, the participants were categorized into two groups, based on the results of preoperative oral screening, to understand whether they required oral management during the perioperative period. In addition, we categorized the patients’ age into three age groups: 20–64, 65–74 (young–old), and ≥74 years (old–old). Second, the chi-squared test and residual analysis were employed to determine any significant differences between the necessary or not necessary groups. Using a logistic regression model, both odds ratios (ORs) and 95% confidence intervals (CIs) were calculated. The groups (oral management necessity vs. no necessity) were used as dependent variables. Independent variables were selected at *p* < 0.05 in the chi-squared test for each variable. Age groups, sex, family dentist oral, and oral examination categories were added as independent variables to the multivariate analysis (forced injection method). We used the Hosmer–Lemeshow test to evaluate the goodness-of-fit of logistic regression models. *p* value of <0.05 was considered statistically significant. Regarding the post sample size calculation, power analysis was conducted. Analyses were performed using the SPSS version 26.0 software program (IBM Corp., Armonk, NY, USA).

## 3. Results

### 3.1. Participants’ Characteristics

Table 2 shows the sample size, sex, age, and the clinical department in which the patients underwent surgery. Overall, 1081 patients (329 men and 752 women) with an average age of 55.9 ± 17.5 (mean ± SD) years were included for analyses. Gynecological patients were the most common, accounting for 38% of the total cohort. Most male patients (49%) had undergone orthopedic surgery.

### 3.2. Comparison of Variables among the Three Groups by Age Group

Table 3 depicts the comparison of patient characteristics and the results of oral screening among the three groups by age group. The chi-squared test and residual analysis revealed the following: (1) females significantly tended to be in the <65 years age group than males, and males significantly tended to be in the 65–74 and ≥75 years age groups than males; (2) patients who had a family dentist significantly tended to be in the <65 years age group, and the patients who did not had a family dentist significantly tended to be in the 65–74 and ≥75 years age groups; (3) patients who did not have tooth mobility, isolated tooth, or inadaptation of denture significantly tended to be in the <65 years age group, and the patients who had such tooth or inadaptation of denture tended to be in the 65–74 and ≥75 years age groups; (4) patients who did not have severe dental caries or poor oral hygiene significantly tended to be in the <65 years age group, and the patients who had such tooth or poor oral hygiene tended to be the ≥75 years age group.

“Poor oral hygiene” was the most frequent (24%) cause in the oral examination categories, whereas “Oral dryness” was the least frequent (0.6%). “Tooth mobility,” “Isolated tooth,” “Severe dental caries,” “Inadaptation of denture,” and “Poor oral hygiene” were statistically more common among the older patients.

### 3.3. Oral Management Necessity during the Perioperative Period

Table 4 compares the oral status between the two groups, stratified by the necessity of oral management after oral screening. The proportion of older people was higher in the oral management necessity group than in the other groups (*p* < 0.001), with a comparatively greater number of men (*p* < 0.001). The patients without a family dentist were more likely to have oral management necessity (*p* = 0.018). Residual analysis revealed that the <65 years age group significantly tended to be in the “Not need” category, and other two groups significantly tended to be in the “Necessity” category. In the oral management necessity group, 65% of the patients had “Poor oral hygiene,” and the cases of “Gingival inflammation” and “Tooth mobility” corresponded to 42% and 39%, respectively. In each oral examination category, the proportion of patients in the oral management necessity group was higher than those in the other groups (*p* < 0.001, respectively).

After the judgment of oral management necessity, of the 188 patients who were recommended to visit dentists, 124 planned to visit the perioperative care center at the Kyushu University Hospital and 60 wanted to go to a family dentist, but four patients did not intend to visit either.

### 3.4. Factors Associated with Oral Management Necessity during the Perioperative Period

Table 5 shows the results of logistic regression analysis. Older people showed about twice the OR compared with those aged <64 years. Sex and the existence of a family dentist were not associated with oral management necessity. Except for isolated tooth and inadaptation to the denture, each oral examination category was independently associated with the need for oral management. The highest odds ratio was tooth mobility (OR: 21.476; 95% CI: 11.462–40.239; *p* < 0.001), followed by oral dryness (OR: 8.584; 95%: 1.031–71.436; *p* < 0.047). The accuracy of discrimination was 90.5%, whereas the Hosmer–Lemeshow test found the model fit to be acceptable (*p* = 0.319).

### 3.5. Post-Hoc Analysis

Post-hoc analysis was performed with tha sample size presented in Table 3 and α = 0.05; the lowest effect size was 0.167 in the relationship between oral management necessity and family dentist. In the other categories, the range of effect size was 0.361–0.750, which was medium or high. The lowest power (1-β) was 0.995 in the relationship between oral management necessity and family dentist.

## 4. Discussion

Our study revealed the frequency of problematic oral status preoperatively, and 188 patients (17%) were found to need oral management during the perioperative period, owing to their oral status. Relevant past studies have focused on oral hygiene before surgery [18,19] or have targeted older patients only [12]. To the best of our knowledge, there are only a few studies that have targeted a large number of patients with a comprehensive evaluation of their oral status before surgery. Our study examined which preoperative oral status affected the judgment of oral management necessity and found it to be “tooth mobility” in our hospital. Our results will be useful in considering perioperative oral management in the future. However, the effectiveness of the oral screening tool used in this study remains unclear because the relationship between the results of oral screening and postoperative complications was not investigated in the present study. Further studies are necessary to define categories, investigate the relationship with an oral examination in detail along with the postoperative complications, and reveal the effectiveness of the oral screening tool used during the perioperative period.

There have been only a few studies on preoperative oral screening. For instance, Sekiya et al. [11] evaluated nine categories (i.e., teeth movement, gingiva inflammation, dental calculus, dental plaque, tongue, xerostomia, halitosis, dysphagia, and trismus) and recommended patients to visit an oral surgery clinic if there was even one problematic finding. As a result, the number of patients who underwent oral screening and required oral management was found to range from 16.4% to 26.5%. Although their target population was only cancer surgery patients and there were differences in oral categories in our study, these ratios of patients requiring oral management were similar. However, past studies did not reveal the applicability rate of each category. Therefore, the finding that “poor oral hygiene” was the most frequent and “tooth mobility” was the greatest concern is very meaningful. On the other hand, patients with oral dryness were very few (0.6%) in this study. This is probably because the standard of dryness was strict (score = 2 by OHAT). Because the relationship between oral dryness and pneumonia was reported previously [20], we paid attention only when considering a review of the standards. In addition, we found that the older patients needed more extensive oral management. These results are supported by past reports that older people have more oral troubles [14] and are at a greater risk of the onset of postoperative pneumonia [11].

In our study, no association between oral management necessity and family dentist was noted. We only asked whether patients had family dentistry; we should have instead asked whether they undergo oral care maintenance regularly, or when did they last visit their dentist. Cooperation with a family dentist is important, and we have to provide them with information about the patients’ condition or necessary dental treatment [10,11,21]. Particularly, the manpower of dentists may be insufficient in private hospitals, requiring medical cooperation. Therefore, we believe that it is necessary to ask whether patients have a family dentist for better oral management.

Preoperative oral screening was not conducted at any predetermined opportunity because the patients received it on the day when the treatment policy of surgery was decided. In other words, some of the patients were screened a few months before the surgery, whereas others were screened a few days after. Moreover, some patients received chemotherapy until just before oral screening. Therefore, the results of oral screening were not the same as those just before surgery. We need to be cautious when interpreting the present results and conduct analyses of the relationship between oral screening and postoperative complications in future studies.

For better oral management, preoperative oral screening should be conducted as soon as possible. After the oral screening, patients could visit dentists to improve their oral status [22]. Moreover, if there is sufficient time until surgery, one can plan tooth extraction while considering the prevention of infective endocarditis and bloodstream disease [5,8,23]. A previous study reported that the reservoirs of oral infection should be removed at least two weeks before a surgical procedure [11]. If we want to ensure recovery of the oral function using prosthetic treatment before surgery, sufficient time may be needed. Nevertheless, it may be important that the oral status of patients be managed by family dentists on a daily basis.

The present study has some limitations. First, all participants were Japanese and underwent oral screening before surgery at the Kyushu University Hospital. Moreover, they belonged to specific clinical departments, which is important to consider, because other general surgeries and transplant patients have systematic oral management supported by the Japanese insurance system. For example, a previous study reported that patients with esophageal cancer have a high incidence of severe periodontitis [24]. Not including such patients may limit the ability to extrapolate the findings to the general patients. Second, because this study used retrospective data and had staff replacements in 16 months, it was difficult to perform calibration among the entire staff despite using a unified manual for oral screening. Therefore, there may be differences in the results of screening, depending on the examiners. In addition, there was no cut-off point for oral management necessity in our screening method because the examiners evaluated the oral status based on checklists and interviews and judged the need for oral management. Thus, this preoperative oral screening method may not be available to non-dental professionals.

We might need to perform analysis of postoperative management of cases that agreed or refused to receive oral management during the perioperative period. However, in our study, only 4 of 188 who patients who were recommended to visit dentists refused to receive the oral management. Therefore, we could not analyze their data, owing to the very small sample size. Additional studies using larger sample size and diverse clinical departments or joint research with other facilities are needed to confirm the relationship between the results of preoperative oral screening and postoperative complications. Further development and assessments of preoperative oral screening tools will provide us with new perspectives for more effective oral management during the perioperative period.

## 5. Conclusions

The results of the present study revealed the high frequency of cases of poor oral hygiene in preoperative oral screening categories. In addition, tooth mobility was found to be strongly associated with oral management necessity, as judged by dentists during the perioperative period.

## Figures and Tables

**Table 1:** Assessments of three oral examination categories in this study.

Category	0 = Healthy	1 = Changes	2 = Unhealthy
Gums and tissues	Pink, moist, smooth, no bleeding	Dry, shiny, rough, red, swollen, one ulcer/sore spot under dentures	Swollen, bleeding, ulcers, white/red patches, generalized redness under dentures
Saliva	Moist tissues, watery and free-flowing saliva	Dry, sticky tissues, little saliva present, resident thinks they have a dry mouth	Tissues parched and red, very little/no saliva present, saliva is thick, resident thinks they have a dry mouth
Oral cleanliness	Clean and no food particles or tartar in mouth or dentures	Food particles/tartar/plaque in 1or 2 areas of the mouth or on a small area of dentures or halitosis (bad breath)	Food particles/tartar/plaque in most areas of the mouth or on most dentures or severe halitosis (bad breath)

These three categories are some excerpts from Oral Health Assessment Tool.

**Table 2:** Participants’ characteristics.

Variable		Total (*n* = 1081)	Male (*n* = 329)	Female (*n* = 752)
Age (years)		55.9 ± 17.5	59.6 ± 17.8	54.3 ± 17.1
Age group	<65	680 (63)	170 (52)	510 (68)
	65–74	225 (21)	90 (27)	135 (18)
	≥75	176 (16)	69 (21)	107 (14)
Clinical department	Gynecology	410 (38)	0 (0)	410 (55)
	Dermatology	132 (12)	73 (22)	59 (7.8)
	Neurosurgery	2 (0.2)	2 (0.6)	0 (0)
	Orthopedic Surgery	333 (31)	161 (49)	172 (23)
	Plastic Surgery	72 (6.7)	22 (6.7)	50 (6.6)
	Ophthalmology	131 (12)	71 (22)	60 (8.0)
	General Surgery	1 (0.1)	0 (0)	1 (0.1)

Data are shown as mean ± SD or number (%).

**Table 3:** Comparison of variables among the three study groups by age groups.

Variables		Total (*n* = 1081)		Age Group		*p* Value *
			<65 (*n* = 680)	65–74 (*n* = 225)	≥75 (*n* = 176)	
Sex	Male	329 (30)	170 (25)	90 (40)	69 (39)	<0.001
	Female	752 (70)	510 (75)	135 (60)	107 (61)	
Family dentist	Have	767 (71)	457 (67)	174 (77)	136 (77)	0.002
	No	314 (29)	223 (33)	51 (23)	40 (23)	
Tooth mobility	Have	95 (8.8)	34 (5.0)	36 (16)	25 (14)	<0.001
Isolated tooth	Have	125 (12)	54 (7.9)	35 (16)	36 (21)	<0.001
Severe dental caries	Have	101 (9.3)	51 (7.5)	24 (11)	26 (15)	0.009
Tooth pain	Have	52 (4.8)	33 (4.9)	14 (6.2)	5 (2.8)	0.290
Inadaptation of denture	Have	52 (4.8)	14 (2.1)	23 (10)	15 (8.5)	<0.001
Gingival inflammation	Have	112 (10)	64 (9.4)	28 (12)	20 (11)	0.386
Oral dryness	Have	7 (0.6)	3 (0.4)	2 (0.9)	2 (1.1)	0.520
Poor oral hygiene	Have	254 (24)	140 (21)	56 (25)	58 (33)	0.002

Values are number (%). * Pearson’s chi-square test among three groups by age groups.

**Table 4:** Comparison of variables between two groups by oral management necessity during the perioperative period.

Variables		Oral Management Necessity		*p* Value *
		Necessity (*n* = 188)	Not Need (*n* = 893)	
Age group	<65	84 (45)	596 (67)	<0.001
	65–74	56 (30)	169 (19)	
	≥75	48 (25)	128 (14)	
Sex	Male	86 (46)	243 (27)	<0.001
	Female	102 (54)	650 (73)	
Family dentist	Have	120 (64)	647 (72)	0.018
	No	68 (36)	246 (28)	
Tooth mobility	Have	74 (39)	21 (2.4)	<0.001
Isolated tooth	Have	62 (33)	63 (7.1)	<0.001
Severe dental caries	Have	58 (31)	43 (4.8)	<0.001
Tooth pain	Have	23 (12)	29 (3.2)	<0.001
Inadaptation of denture	Have	21 (11)	31 (3.5)	<0.001
Gingival inflammation	Have	78 (42)	34 (3.8)	<0.001
Oral dryness	Have	5 (2.7)	2 (0.2)	<0.001
Poor oral hygiene	Have	122 (65)	132 (15)	<0.001

Values are number (%). * Pearson’s Chi-square test.

**Table 5:** Adjusted odds ratios and 95% confidence intervals for oral management necessity during the perioperative period.

**Variables**		**Odds Ratio**	**95% Cl**	***p* Value**
Age group	<65	1 (reference)		
	65–74	1.931	1.122–3.323	0.017
	≥75	2.049	1.150–3.649	0.015
Sex	Male	1 (reference)		
	Female	0.669	0.421–1.063	0.089
Family dentist	Have	1 (reference)		
	No	1.193	0.727–1.959	0.485
Tooth mobility	Have	21.476	11.462–40.239	<0.001
Isolated tooth	Have	1.531	0.825–2.840	0.177
Severe dental caries	Have	2.940	1.557–5.553	0.001
Tooth pain	Have	3.014	1.250–7.265	0.014
Inadaptation of denture	Have	1.487	0.655–3.376	0.343
Gingival inflammation	Have	8.011	4.472–14.350	<0.001
Oral dryness	Have	8.584	1.031–71.436	0.047
Poor oral hygiene	Have	4.462	2.754–7.229	<0.001

Model fit (forced injection method): Hosmer–Lemeshow test (*p* = 0.319), and the accuracy of discrimination was 90.5%. Dependent variable: oral management necessity (0: necessity, 1: not need). Independent variables: Age groups, sex, family dentist, and oral examination categories.

## Data Availability

The data that support the findings of this study are available on request from the corresponding author, S.M. The data are not publicly available due to restrictions e.g., their containing information that could compromise the privacy of research participants.
